# The effect of postoperative complications on health-related quality of life and survival 12 years after coronary artery bypass grafting – a prospective cohort study

**DOI:** 10.1186/s13019-021-01527-6

**Published:** 2021-06-14

**Authors:** Matti Hokkanen, Heini Huhtala, Jari Laurikka, Otso Järvinen

**Affiliations:** 1 Department of Cardio-Thoracic Surgery, Tays Heart Hospital, Tampere University Hospital, Tampere, Finland; 2 Faculty of medicine and health technology, Tampere University, Tampere, Finland; 3 Faculty of Social sciences, Tampere University, Tampere, Finland

**Keywords:** Coronary artery bypass, Quality of life, Outcomes, Survival, Postoperative complication

## Abstract

**Background:**

Despite the steady improvements in survival and operative safety, postoperative complications still remain a significant cause of morbidity and mortality after coronary artery bypass grafting (CABG). However, less is known on the impact of postoperative complications on health-related quality of life (QoL). The main objective of our study was to investigate the impact of postoperative complications on long-term QoL and survival after CABG surgery.

**Methods:**

Data of 508 patients, who underwent isolated CABG was prospectively collected. The RAND-36 Health Survey (RAND-36) was used to evaluate patients’ QoL status preoperatively, 1 year and 12 years after the surgery. Predefined postoperative complications were reported during primary and secondary hospital stay. QoL and survival analysis were performed primarily on three patient groups: patients with and without complications and patients with major adverse cardiac and cerebrovascular events (MACCE).

**Results:**

In total 205(40%) of 508 patients had at least one postoperative complication and 73 (14%) experienced MACCE. Patients’ thirty-day, 1-year and 10-year survival rates were, 99, 98, 84% without complications, 97, 95, 72% with complications, and 90, 89, 64% with MACCE, respectively (log-rank *p* < 0.001). Patients without complications showed significant(*p* < 0.05) improvements in seven and patients with complications in five out of eight RAND-36 QoL dimensions. All patient groups showed significant improvements in RAND-36 summary scores compared with preoperative values. Patients with complications and especially with MACCE had more profound decline in their RAND-36 summary scores while patients without complications maintained their health status best.

**Conclusions:**

Despite the constant deterioration, both patients with and without complications showed improvements even 12 years after CABG compared with preoperative state. Postoperative complications and especially MACCE were associated with impaired long-term QoL.

## Background

Coronary artery bypass grafting (CABG) has remained the most commonly performed cardiac surgery procedure over the past decades [1]. The main goal of the surgery is to improve survival and health related quality of life (QoL) [2]. Despite the fact that the profile of patients undergoing isolated CABG surgery has changed towards higher age with increased frequency of preoperative co-morbidities [3, 4] mortality and severe morbidity after CABG have even decreased over past decades [1, 5]. However, the life-expectancy of older and more moribund patients may be limited due to natural causes which emphasizes the importance of QoL as indication for surgery.

Whilst CABG surgery’s safety and efficacy in treating patients with coronary artery disease (CAD) is well-established, the complications of the surgery still remain a burden causing mortality, morbidity and decrease in QoL [5]. The outcomes of CABG surgery are mainly studied in terms of survival benefit and relief from cardiac related symptoms. Yet, there is a lack of long-term data on the effects of postoperative complications on QoL. In this prospective cohort study we evaluated the impact of postoperative complications on long term health related QoL and survival 12 years after CABG procedure. Special interest was focused on the impact of major adverse cardiac and cerebrovascular events (MACCE) on postoperative quality of life.

## Methods

The original data on operations were obtained from Tampere University Hospital between May 1999 and November 2000. The study was approved by the institutional review board of Tampere university and all participating patients gave informed consent. The study cohort included 508 patients who had CABG surgery electively or urgently. Extensive medical data was prospectively collected pre-, per- and postoperatively during primary hospital stay. Majority of patients were discharged to 18 local district hospitals and data from these hospitals were collected by referring physicians and send to authors for further analysis. All predefined complications and outcome events were recorded for joint analysis with the primary hospital data. Statistical Finland provided survival data and dates of death after discharge and also survival data for age-, sex- and hospital-catchment-area-matched controls (1:3).

Total number of patients operated in Tampere University Hospital during study period was 1128. Patients who were emergently operated or unable to complete the survey were excluded. In comparison to 508 included patients, the excluded ones were less often male (402 (65%) vs. 420 (83%) patients *p* < 0.001), were older (mean age 67 vs 62 years *p* < 0.001), were operated more urgently (293 (47%) vs 109 (22%) patients *p* < 0.001), had more often ejection fraction under 50% (166 patients (28%) vs 92 (18%) patients *p* < 0.001), had more often history of stroke (43(6.9%) vs 20(3.9%) patients, *p* = 0.036) or diabetes (119 (19%) vs 74 (15%) patients *p* = 0.47). Preoperative characteristics of the patient groups are shown in Table 1.

**Table 1:** Demographic data and clinical characteristics of the 1128 patients operated during the study period

	Total number of included patients = 508			Total number of excluded patients = 620
	No complications	Any complications		
	MACCE			
	Number of patients(%)	Number of patients (%)	Number of patients (%)	Number of patients
Total number	303 (60)	205 (40)	73 (14)	620
Mean age, years [SD]	60.5 [9.1]	65.0 [9.0]	67.2 [8.7]	66.5 [9.5]
Male	255 (84)	165 (80)	57 (78)	204
Operative priority				
Elective	248 (82)	151 (74)	55 (75)	288
Urgent or emergency	55 (18)	54 (26)	18 (25)	293
Euroscore 1 mean [SD]	2.1 [2.0]	3.5 [2.7]	4.1 [2.5]	4.65[3.0]
Ejection fraction <50%	51 (17)	41 (20)	19 (27)	166
Left main stem >50%	55 (18)	52 (25)	15 (21)	140
Redo surgery	8 (3)	18 (9)	8 (11)	27
History of stroke	5 (2)	15 (7)	5 (7)	43
History of TIA	8 (3)	8 (4)	1 (1)	35
History of acute myocardial infarction	130 (43)	84 (41)	30 (41)	178
impaired kidney function krea > 141mmol	2 (1)	5 (2.5)	2 (3)	18
Off-pump surgery	45 (15)	11 (5)	4 (6)	58
Diabetes	36 (12)	38 (19)	8 (11)	119
Obesity	55 (18)	45 (22)	13 (20)	143
History of general ASO	18 (6)	19 (9)	9 (12)	52
COPD	15 (5)	18 (9)	3 (4)	49
Persistent AF	6 (2)	11 (5)	1 (1)	20
Hyperlipidemia	223 (74)	149 (73)	50 (69)	443
Hypertension	147 (49)	121 (59)	45 (62)	346

*MACCE* Major adverse cardiac and cerebrovascular event, *TIA* Transcient ischemic attack, *ASO* Arteriosclerosis obliterans, *COPD* Chronic obstructive pulmonary disease, *AF* Atrial fibrillation

**Fig. 1:**
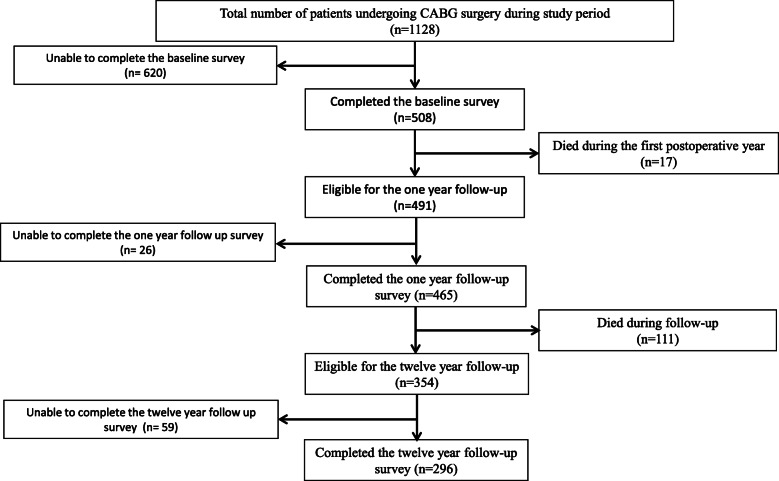
Consort diagram of patients included in the study

First, a baseline QoL questionnaire was given to patients 1 day before surgery. Follow-up questionnaires were mailed to patients 1 year and 12 years after surgery. During the first postoperative year 17 patients (3%) died and 465 (95%) of 491 surviving patients returned the one-year follow-up questionnaire, mean follow-up time being 12.6 months (SD 1.2). Those 26 patients who did not complete the one-year follow-up survey, were younger (median age 54 vs. 63 years *p* = 0.006) in comparison to 465 patients who completed it. Otherwise the groups were closely similar in terms of sex, Euroscore 1 risk sum, priority of operation or NYHA glass. Next, the same follow-up questionnaire was mailed to patients 12 years after the surgery median follow-up time being 11.8 years (SD 0.48). Two hundred and 96 patients had died during this second follow-up period and 296 (84%) of the 354 surviving patients completed the follow-up questionnaire. There were no statistically significant differences in preoperative characteristics between the patients who completed and who didn’t complete the 12 year follow-up surveys. The enrolment of the patients is summarized in Fig. 1.

The main focus of the study was to evaluate the effect of postoperative complications occurring after CABG operation during primary and secondary hospital stay on patients’ QoL and survival. The complications were predefined and included: 1) Re-sternotomy (due to bleeding or low-out put syndrome), 2) mediastinitis (deep sternal wound infection requiring debridement), 3) sternal or graft harvest site wound infection (symptomatic collection of pus or dehiscence of the wound requiring debridement), 4) gastrointestinal complication (radiologically and clinically verified cholecystitis, pancreatitis, ileus or acute mesentery ischemia), 5) stroke (clinically and radiologically verified by experienced neurologist), 6) acute kidney failure (in need of dialysis or increase of serum creatinine level more than 2-fold from baseline value), 7) pneumonia (causing clinical symptoms and radiologically verified), 8) pulmonary embolism (radiologically verified) 9) prolonged ventilatory support (> 36 h), 10) pleural effusion (radiologically verified and requiring treatment), 11) respiratory failure (in need of intubation or mechanical ventilation support), 12) atrial fibrillation (newly diagnosed by ECG requiring cardioversion or permanent anticoagulation), 13) ventricular tachycardia or bradyarrhythmia (requiring intensive care unit (ICU) or cardiac care unit (CCU) stay), 14) low out put syndrome (requiring the use of intra aortic balloon or prolonged need for inotropic medication), 15) Perioperative myocardial infarction (including new Q wave in electrocardiogram and a peak CK-MB level of more than 150 μmol/l), 15) postpericardiotomy syndrome (including fever, new or worsening pericardial and pleural effusion) and 16) delirium (deterioration of neuropsychological status requiring medication). Each patient within complication group could have had one or more postoperative complication. Major adverse cardiac and cerebrovascular events (MACCE), including re-sternotomy, mediastinitis, stroke, acute kidney failure and perioperative myocardial infarction, were also analyzed as an independent group. QoL and survival of patients with MACCE were compared with patients without MACCE, which included patients with other complications than MACCE or no complications.

The primary outcome of the study was health-related QoL which was assessed with Finnish adaption of RAND 36 generic health –related QoL scale. RAND-36 is a validated and widely used scale for which there are reference values available for Finnish population [6]. It comprises scores for eight different dimensions of health-related QoL: 1) general health, 2) physical functioning, 3) role functioning/physical 4) bodily pain, 5) emotional well-being, 6) role functioning/emotional 7) social functioning and 8) energy [7, 8]. The scores for each domain range from 0 to 100, 0 being the poorest and 100 the best possible health status. In addition, two summary scores can also be used: the physical component summary (PCS) including the mean value of physical sub-scales, whilst mental component summary (MCS) equals subscales (5–8) reflecting psychosocial health status [9–11].

Patients and outcome variables are mostly expressed as proportions of the total (in percentage), and continuous variables as means with standard deviation (SD). Categorical variables between the groups were compared by using chi-square test. Continuous variables were analysed with Mann-Whitney test. Baseline, 1 year and 12-year follow-up scores for QoL were compared using paired-samples t-test. PCS and MCS scores were compared in three time points with variance analysis for repeated measures. The Kaplan-Meier survival curves of assessed patient groups were compared using log-rank test. Statistical analyses were performed with SPSS 20.0 for Windows (IBM).

## Results

Three hundred and ninety operations out of the 508 included in the analysis were performed electively and 110 (22%) urgently. Eighty-two percent of all patients were male and patients age varied between 34 to 92 years (median 63). Preoperative characteristics of all patients are shown in Table 1. In this study cohort, patients with complications were older (median age 66 vs. 60 years *p* < 0.001), had higher Euroscore 1 risk sum (median 3 vs. 2 *p* < 0.001), had more often history of stroke (7.3% vs. 1.7% *p* = 0.002), hypertension (59.0% vs 49.0% *p* = 0.02), persistent atrial fibrillation (5.0% vs. 2.0% *p* = 0.037), diabetes (19 vs. 12% *p* = 0.04) and were more often operated as a redo surgery (9.0% vs 3.0% *p* = 0.002). Otherwise both groups were closely similar in terms of preoperative characteristics. Patients with MACCE were older (median 69 vs. 62 years *p* < 0.001), had higher Euroscore 1 risk sum (median 4 vs 2 *p* < 0.001) and were more often operated as a redo surgery (11% vs. 4.0% *p* = 0.002) in comparison to patients without MACCE.

The types and frequencies of complications within the patient cohort are described in Table 2. In the total cohort of 508 patients, 205 patients (40%) experienced at least one of the complications included in our analysis. Seventy-three patients (14%) had MACCE during postoperative period.

**Table 2:** Number of patients with intra- and postoperative complication following CABG surgery in 508 patients included in the study^a^

	Total cohort *N*=508	1 year *n*=465	Responders at 12 years *n*=296
Complication			
No	303	276	193
Yes	205	189	103
MACCE	73	62	33
Re-sternotomy	15	11	6
Bleeding	13	10	5
Low out put syndrome	2	1	1
Mediastinitis	9	8	2
Stroke	15	13	6
Renal failure or dialysis	19	14	6
Perioperative myocardial infarction	22	20	14
Wound infection	69	66	32
Superficial sternal wound infection	12	12	6
Graft harvest site	57	54	26
Gastrointestinal complication	4	4	2
Pneumonia	9	9	4
Atrial fibrillation	19	19	12
Respiratory failure	12	10	7
Tachycardia or bradyarrhythmia	47	42	24
Pleural effusion	24	22	13
Delirium	20	19	9
Low output syndrome	12	7	4
Prolonged postoperative ventilatory support	12	11	5
Postpericardiotomy syndrome	10	10	4
Pulmonary embolism	7	7	3

^a^ Number of complications may exceed the number of patients when patients had more than one complication

**Fig. 2:**
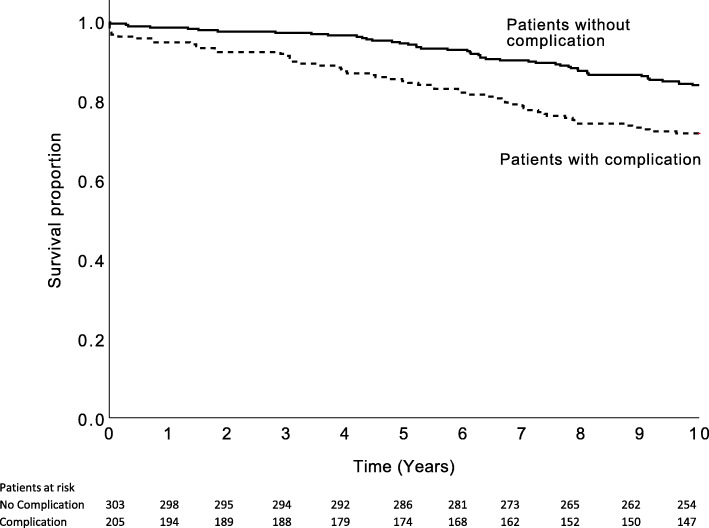
Kaplan-Meier survival curves for patients with and without complications (Log rank < 0.001)

**Fig. 3:**
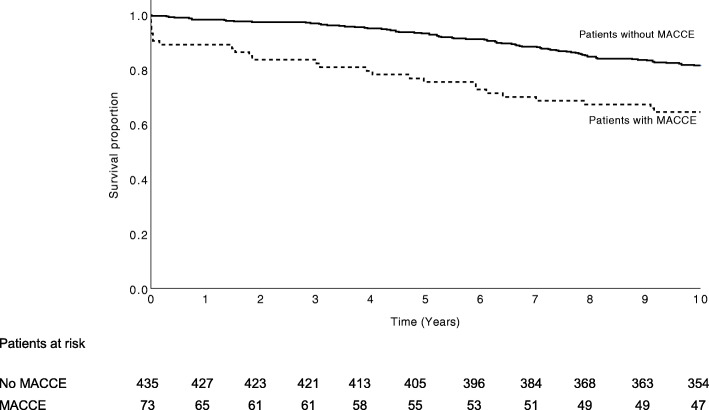
Kaplan-Meier survival curves for patients with and without major cardiac and cerebrovascular events (MACCE) (Log rank < 0.001)

Thirty-day, 1-year and 10-year survival rates were 97% (7 deaths), 95% (11 deaths), 72% (58 deaths) in patients with complication and 99% (2 deaths), 98% (5 deaths), 84% (49 deaths) in patients without complications, respectively. Patients with complications had lower survival rates than patients without complication (log rank *p* < 0.001). Moreover, thirty-day, 1-year and 10-year survival rates were 90% (7 deaths), 89% (8 deaths) and 64% (26 deaths) in patients with MACCE. Hospital-catchment-area matched controls had a 10-year survival rate of 77%. In comparison with age-, sex- and hospital-catchment-area matched controls, only patients without complications had higher ten-year survival. Figs. 2 and 3 show Kaplan-Meier survival curves for all patient groups.

The RAND- 36 QoL mean scores in patients with and without complications and MACCE are shown in Table 3. The studied patient groups had inferior preoperative baseline QoL values when compared to Finnish general population. One year after surgery, all groups showed higher health scores in all eight QoL dimensions, but in the later follow-up period their scores deteriorated. Twelve years after bypass grafting, patients without complications maintained significant improvements in all but one RAND-36 dimensions (in general health), whereas patients with complications showed statistically significant long-term improvements in all but three different dimensions (in general health, emotional role functioning and energy). Furthermore, patients with MACCE maintained higher QoL score only in four RAND- 36 dimensions (in physical functioning, physical role functioning, emotional role functioning and pain). Patients with no complications showed higher QoL scores in all RAND-36 dimensions in comparison with age- and sex-matched reference values. The patients with complications and MACCE had elevated health status only in few QoL dimensions, when compared with reference population scores. Above all, changes in physical role functioning and pain were most notable in all patient groups.

**Table 3:** Quality of life scores (RAND 36) for survivors and general population

QOL category	Group	Baseline	1-year follow-up	12-year follow-up	General population^a^	Change^b^	*p*-values
		*n*=508	*n*=465	*n*=296			
General health	No complication	53.3	58.3	56.8	46.7	3.5	0.22
	Complication	48.8	52.1	48.8		0	0.20
	MACCE	48.9	54.3	52.6		3.7	0.70
Physical functioning	No complication	50.4	77.4	68.9	58.7	18.5	<0.001
	Complication	43.8	68.1	57.6		13.8	<0.001
	MACCE	42.7	68.9	54.0		11.3	0.011
Social functioning	No complication	67.7	83.5	81.5	75.7	13.8	<0.001
	Complication	64.7	77.5	68.9		4.2	<0.001
	MACCE	62.3	77.9	64.6		2.3	0.44
Role functioning/physical	No complication	22.9	60.2	56.8	39.1	33.9	0.04
	Complication	18.2	45.5	40.2		22	<0.001
	MACCE	15.8	44.8	34.9		19.1	0.002
Role functioning/emotional	No complication	43.5	66.0	66.9	51.3	23.4	<0.001
	Complication	37.7	62.0	47.4		9.7	<0.001
	MACCE	35.8	65.5	42.8		7	<0.001
Emotional well-being	No complication	73.3	77.5	78.9	71.8	5.6	0.001
	Complication	72.2	74.9	70.4		-1.8	0.246
	MACCE	72.0	74.7	69.2		-2.8	0.366
Energy	No complication	58.3	67.9	66.8	59.4	8.5	<0.001
	Complication	54.0	63.5	54.8		0.8	0.42
	MACCE	53.4	64.3	53.4		0	0.83
Pain	No complication	50.6	76.4	74.6	63.5	24	<0.001
	Complication	44.7	72.5	64.9		20.2	<0.001
	MACCE	44.6	73.8	64.1		19.5	<0.001

^a^Age and sex-adjusted reference values for 12-year follow-up scores.^b^Between 12-year and baseline scores. ^c^Paired-samples t test between the baseline and 12-year follow-up scores

Both patients with and without complications showed statistically significant improvements in RAND-36 PCS (*p* < 0.001) and MCS (*p* < 0.001) summary scores 1 and 12 years after CABG surgery compared with their preoperative scores (Table 4). Both groups had high physical and mental summary scores 1 year after surgery. In the late follow-up both PCS and MCS scores were lower. Patients with complications showed deeper decline in their MCS scores (*p* = 0.004) and PCS scores (*p* = 0.052) whilst patients without complications maintained their perceived QoL score status better. However, these groups showed elevated QoL score status even 12 years after the surgery when compared with their preoperative values. Patients with MACCE improved their PCS (*p* < 0.001) and MCS(*p* = 0.004) scores 1 and 12 years after surgery. However, patients with MACCE had lower PCS (*p* = 0.02) and MCS(*p* = 0.069) scores compared with patients with no MACCE 1 and 12 years after the CABG surgery.

**Table 4:** Mean change in RAND-36 physical component summary (PCS) and mental component summary (MCS) scores of the study patients in relation to occurrence of postoperative complications

					*P*-values^a^		
	Group	Baseline	1 year	12 years	Interaction between time*group	Time	Group
PCS scores	No complication	47.1	70.5	64.9	0.052	<0.001	<0.001
	Complication	41.3	63.3	53.6			
	No MACCE	45.8	68.8	62.0	0.505	<0.001	0.02
	MACCE	39.2	61.1	52.5			
MCS scores	No complication	62.7	76.0	74.2	0.004	<0.001	0.001
	Complication	58.0	72.2	61.6			
	No MACCE	61.5	75.1	70.8	0.301	0.004	0.069
	MACCE	57.1	70.7	61.5			

^a^ Variance analysis for repeated measures

## Discussion

Under the past few decades, the risk profile of patients undergoing isolated CABG has significantly evolved towards higher age with more preoperative comorbidities [4]. Furthermore, CABG has remained the most commonly used treatment for high risk patients with complex CAD [12, 13]. Despite the steady improvements in survival and operative safety, postoperative complications still cause significant morbidity and suffer among operated patients [5, 14]. The impact of postoperative complications is mainly evaluated in survival [14, 15] and economical [16] perspectives. However, the effect of postoperative complications on patients’ quality of life in long term is hitherto unclear. In this prospective cohort study, we evaluated the impact of postoperative complications on long-term Qol and survival. Special interest was focused on the impact of major adverse cardiac and cerebrovascular events on patients` postoperative QoL health-status.

The main finding of our study is that despite the on ongoing deterioration, patients with and without complications and MACCE had higher QoL score status at 1-year and even 12-years after CABG surgery. However, patients without complications maintained their perceived QoL best whereas patients with complications had slightly lower QoL scores 1 year and as long as 12 years after the surgery.

The effect of severe postoperative complications on QoL has remained controversial in previous studies. Major complications have shown to cause impaired postoperative QoL after CABG surgery [17–19]. Peric and colleagues found postoperative complications to be an independent predictor of impaired QoL 6 months after CABG surgery [20]. Moreover, Herlizt et al. demonstrated that postoperative complications such as prolonged stay at ICU, prolonged ventilator time and postoperative use of inotropics predict inferior QoL even after 15 years of after CABG operation [21]. On the contrary, Karhunen and colleagues reported that the long-term QoL of the patients, who survived after emergency re-sternotomy and resuscitation, was similar in comparison to patients without major postoperative complications [22]. In addition, Williams and co-authors found that 83% of patients, who had survived after CABG and related prolonged ICU stay had normal QoL 2 years after surgery [23]. We found that patients with major complications had lower PCS and MCS summary scores at 1 year and 12 years’ follow-up and these patients had deeper decline in their QoL status in comparison to patients without MACCE. However, encouragingly, patients with MACCE showed elevated QoL summary scores even 12 years after the surgery compared with preoperative values.

The incidence of postoperative complications after CABG surgery ranges between 30 and 40% depending on the number of different complication included in the studies [14, 15, 20]. These findings are in line with the incidence of postoperative complication, 40%, represented in our study cohort. The mechanisms behind complications causing impaired QoL can only be speculated upon. Firstly, postoperative complications often prolong primary hospital stay [14], and can cause recurrent hospital readmissions [14, 24] which may delay and interfere with the patients’ rehabilitation progress. Additionally, major complications and prolonged ICU stay can exposure patients to excessive stress reactions which may cause impaired mental and physical QoL [25]. Moreover, postoperative complications may deteriorate patients’ QoL status by causing long lasting local symptoms and physical deficit, such as chronic sternal pain after mediastinitis [26] or physical disability after stroke [27].

A wide range of health measurement tools have been developed to assess health related quality of life, of which Nottingham Health profile (NPH) [28], RAND-36 item health survey [7], the Medical Outcomes study short form (SF36) [8] and EuroQoL (EQ-5D) [29] are most commonly used. We selected the Finnish version of RAND-36 health surveys since it has been carefully validated and contains age and sex-adjusted reference values for the Finnish general population. These reference values originate from the studies of randomly selected population samples from The Finnish Population Register [6].

There are several limitations in our present study. Firstly, all emergently operated patients and those who were unable to complete the baseline survey were excluded from the study. This selection bias may limit the interpretation of the result mainly to electively and urgently treated patients. Yet, the study design was prospective and the follow-up of the study cohort was complete in 95 and 84% of patients at 1 year and 12 years, respectively, which partly reduces the impact of selection. Secondly, the study is possibly affected by survival bias since patients who survived over 12 years after CABG surgery with or without complications might have had better physical and mental health status than those, who died earlier. This could partly explain why our study population showed higher QoL scores in comparison to age- and sex-adjusted reference population. Thirdly, in addition to postoperative complications, other, non-cardiac medical comorbidities and adverse health events are shown to have a negative impact on patients’ health status after CABG surgery, especially in the long term follow-up [30]. Therefore we compared patients’ QoL scores with reference values of Finnish general population.

## Conclusions

Despite of ongoing decline 12 years after CABG surgery, both patients with and without complications showed steady improvements in their health-related QoL when compared with the preoperative scores. However, the patients with complications and especially with MACCE had lower postoperative QoL at 1 year and 12 years after the surgery. Due to far-reaching impact of postoperative complications on patients QoL status, a special focus should be directed towards complication prevention and intensive rehabilitation of patients with postoperative complications after CABG surgery.

## Data Availability

The datasets generated and/or analysed during the current study are not publicly available due to hospital data privacy protocol but are available from the corresponding author on reasonable request.

## Abbreviations

AF: Atrial fibrillation
ASO: Arteriosclerosis obliterans
CABG: Coronary artery bypass grafting
CAD: Coronary artery disease
CCU: Cardiac care unit
COPD: Chronic obstructive pulmonary disease
EQ-5D: EuroQoL
ICU: Intensive care unit
MACCE: Major adverse cardiac and cerebrovascular events
MCS: Mental component summary
NPH: Nottingham health profile
PCS: Physical component summary
RAND-36: The RAND-36 health survey
SF36: The medical outcomes study short form
TIA: Transcient ischemic attack
QOL: Quality of life
